# Monobutyrin Reduces Liver Cholesterol and Improves Intestinal Barrier Function in Rats Fed High-Fat Diets

**DOI:** 10.3390/nu11020308

**Published:** 2019-02-01

**Authors:** Thao Duy Nguyen, Olena Prykhodko, Frida F. Hållenius, Margareta Nyman

**Affiliations:** Department of Food Technology, Engineering and Nutrition, Kemicentrum, Lund University, PO Box 124, SE-221 00 Lund, Sweden; olena.prykhodko@food.lth.se (O.P.); frida.hallenius@food.lth.se (F.F.H.); margareta.nyman@food.lth.se (M.N.)

**Keywords:** butyrate, lipid metabolism, cecal SCFA, butter, lard, Wistar rat, lactulose/mannitol test, intestinal permeability

## Abstract

Butyric acid has been shown to reduce high-fat diet-related metabolic disturbances and to improve intestinal barrier function due to its potent anti-inflammatory capacity. This study investigates whether a butyric acid ester, monobutyrin (MB) affects lipid profiles and gut barrier function in a dose-response manner in rats fed butter- or lard-based high-fat diets. Four-week-old male Wistar rats were fed butter-based diets containing 0, 0.25, 0.75 and 1.5 MB g/100 g (dry weight basis) or 0.5 glycerol g/100 g, and diets with lard (La) containing 0 and 0.5 MB g/100 g or a low-fat control diet for 3–4 weeks. Lipid profiles in blood and liver tissue, intestinal permeability and cecal short-chain fatty acids were examined. The results showed a dose-dependent decrease in liver total cholesterol for 1.5 MB (*p* < 0.05) and liver triglycerides for 0.75 MB (*p* < 0.05) and 1.5 MB (*p* = 0.08) groups compared to the high-fat control group. Furthermore, a lower excretion of mannitol in urine in the 1.5 MB group indicated improved intestinal barrier function. When MB was supplemented in the lard-based diet, serum total cholesterol levels decreased, and total amount of liver high-density lipoprotein-cholesterol increased. Thus, MB dietary supplementation can be effective in counteracting lipid metabolism disturbances and impaired gut barrier function induced by high-fat diets.

## 1. Introduction

Consuming a diet, high in fat and low in fiber will result in metabolic disorders implicated in a wide variety of diseases associated to the heart and liver, but such a diet composition may also induce inflammation in the colon [1,2,3]. At molecular level, high-fat diets provoke imbalanced homeostasis, stimulating chronic pro-inflammatory stages, primarily initiated in the gut and eventually affecting peripheral organs, even the brain, as shown in animal models [4,5]. In contrast, several studies performed in humans and rats have demonstrated that dietary fiber may counteract the harmful effects caused by high-fat diets [6,7,8]. This is often explained by the short-chain fatty acids (SCFA), especially butyric- and propionic acids, formed by the colon microbiota.

SCFA, the end products derived from bacterial fermentation of dietary fibers, are suggested to play a protective role against high-fat driven disturbances. Indeed, studies in mice have reported that SCFA may strengthen the defense of the gut wall by increasing expression of tight junction proteins [9,10], leading to reduced pro-inflammatory responses caused by translocation of bacterial components such as lipopolysaccharides (LPS) to the circulation [1,4]. Intestinal barrier dysfunction can be detected by measuring levels of tight junction proteins in the gut tissues, LPS in blood or non-metabolizable sugars such as lactulose and mannitol in urine. The latter provides a simple and non-invasive test predicting intestinal permeability [11]. Notably, an increased lactulose/mannitol ratio in urine has been found before establishment of type 1 diabetes [12]. Increased intestinal permeability is also linked to increased serum concentrations of zonulin, a modulatory protein of intercellular tight junctions/intestinal permeability [12] that is related to obesity, inflammatory bowel diseases, and cancers [13].

Besides increasing the nutritional status of the gut, SCFA exert effective roles in lipid metabolism. In fact, addition of SCFA into rat diets leads to a reduction in blood cholesterol concentrations by suppressing hepatic cholesterol synthesis and expression of genes involved in lipid metabolism [14,15]. Particularly, butyrate has been shown to decrease circulating lipids in human intestinal cells by inhibiting production of intestinal lipid transfer proteins [16]. However, our recently published studies demonstrated that it is also possible to improve lipid profiles and attenuate inflammatory markers in rats by supplementing high-fat diets with monobutyrin (MB), a glycerol ester of butyric acid [17]. Furthermore, when supplementing feed given to chickens having problems with too high intake of fiber, a combination of monobutyrin and tributyrin were shown to decrease serum cholesterol [18,19]. Humans are recommended to increase the intake of dietary fiber, especially those that are giving high amounts of butyric acid during colonic fermentation, but it may for some reason be of interest to increase the amount without adding fiber to the diet.

We have previously shown that adding 0.5 MB g/100 g (dry weight basis) to a high-fat diet containing butter results in lowered liver cholesterol levels in association with changes in expression of bile acid related genes and gut microbiota in rats [17]. Based on these results, we hypothesized that increasing levels of MB could have more pronounced effects on lipid profiles and gut barrier function. The aim of this study was (i) to evaluate whether MB could retain similar effects on liver lipid profiles in a dose-dependent range and (ii) whether it is linked with changes in gut permeability and concentrations of SCFA in the hindgut of rats. In some early papers (1959 and 1976, respectively), relatively high amounts of monobutyrin and tributyrin, as measured by gas-chromatography, were determined in butter (0.189 mM in 100 g of extracted fat from butter and 3–4 g/100 g butter, respectively) [20,21], which may interfere with results of the added MB. Thus, lard as another animal-derived source of saturated fat was used in a separate experiment. In that study, we decided (iii) to investigate whether MB also could change lipids in circulating blood, and whether there were any changes and after how long time.

## 2. Materials and Methods

### 2.1. Animals and Diets

This study was approved by the Local Ethical Review Committee for animal research in Lund, Sweden (approval number M 114–15).

Male Wistar rats, weighing 115 g (SEM 1) and being 4-week-old (Taconic, Denmark), consumed high-fat diets containing butter (180 g/kg dry weight, corresponding to 44 Energy %) as described in Supplementary Table S1. Rats were randomly divided into five groups (*n* = 7/group) and given a high-fat-butter control diet (0 MB), or diets supplemented with three doses (0.25, 0.75, and 1.5 g/100 g on a dry weight basis) of MB (Perstorp AB, Sweden), giving the following description of the groups: 0, 0.25, 0.75, and 1.5 MB. Since glycerol has been reported to stimulate cholesterol synthesis in rats and consists 39% of the MB, a group supplemented with 0.5 glycerol g/100 g (VWR, USA) was also included, abbreviated as 0.5 G. The doses of MB chosen were based on the reduced levels of liver total and LDL-cholesterol seen in a previous study [17] and below reported non-toxic levels (27 g of MB/kg body weight/day) in rats [22]. The rats had free access to the assigned diets and water for 3 weeks. However, the feed intake was recorded by weighing feed residues and the amount distributed. Five days before the end of the study, the lactulose/mannitol test was carried out for 24 h. At the end of the study, hepatic portal vein blood, liver and cecum including its content were collected for measurements of lipids (portal vein blood and liver) and SCFA (cecum). Cecum was weighed with and without its content and frozen.

During the dose-response study it was figured out that butter may contain butyric acid in the form of monobutyrin and tributyrin [20,21]. We therefore performed another set of experiment, where the fat source was lard (Dragsbaek, Denmark). This study was designed in the same way as the first one, i.e., the same initial weight of the rats and composition of the high-fat diets and the rats had free access to the diets and water. Rats were randomly divided into 3 groups (*n* = 7/group) fed the following diets: low-fat (LF), high-fat-lard (La) control, and high-fat-lard supplemented with 0.5 MB g/100 g (La + 0.5 MB). At the end of the study, liver and blood (from the hepatic portal vein and aorta) were collected for lipid analyses. Changes in blood lipid levels were also evaluated weekly in the tail vein to figure out if it was possible to measure changes in blood lipids during an on-going experiment. Due to the assumed difficulties to detect differences in lipid content of the circulated lipids in the tail, the experiment was prolonged for another week, to four weeks, before finishing.

Body weight and food intake were tracked weekly in both experiments.

### 2.2. Lipid Analysis in Serum and the Liver

Total cholesterol (esterified and free), triglycerides (TG), low-density lipoprotein- (LDL-) cholesterol, and high-density lipoprotein- (HDL-) cholesterol in portal vein and aortic serum and liver tissues were determined using enzymatic colorimetric kits purchased from Thermo Scientific (Middletown, USA). The blood and liver tissues were collected from anesthetized animals in the following order. First, the abdomen was opened and blood (approximately 2 mL) was collected from the hepatic portal vein into a sterile syringe and transferred into serum collection tubes (Becton Dickinson BD Vacutainer^®^ SST™, Franklin Lakes, NJ, USA) containing sprayed-coated silica. Next, the thorax was opened and blood from the aorta (approximately 2 mL) was collected into another syringe and immediately transferred into the BD tubes. The blood samples were standing for 30 min at room temperature, before centrifuged at 2000 *g* for serum collection. At last, when a cut-off of the heart was performed and the animal was considered being euthanized, the liver was dissected out, weighed, and saved at −40 °C for freeze-drying procedure [23].

Serum samples were assayed directly, while lipids in the freeze-dried livers were extracted in hexane and iso-propanol (ratio 3:2) with 0.005% (*v/w*) 2,6-di-tert-butyl-4-metylphenol, as previously described [17]. The supernatants from the extraction were collected after centrifugation and dried under nitrogen flow at room temperature. The dried extracted lipids were re-dissolved in isopropanol containing 1% Triton X100 (*v/v*) and were then used for lipid determination.

### 2.3. Short-Chain Fatty Acids in Cecal Content

SCFA in cecal contents were analyzed by a gas-chromatographic method developed at our lab by following Zhao et al. [24]. Briefly, cecal contents were extracted in acidified water without using any organic solvents, and centrifuged. The supernatants were injected into a gas chromatographic system (Agilent 6890N GC) equipped with a flame ionization detector (FID) for determination of SCFA. A fused-silica capillary column with a free fatty acid phase and rather water-resistant was used (DB-FFAP 125-3237, J&W Scientific, Agilent Technologies Inc., Santa Clara, CA, USA). The temperature of the injection port and the FID was 200 and 240 °C, respectively. The initial oven temperature was 100 °C and it was kept there for 0.5 min, increased to 180 °C at 8 °C/min and maintained there for 1.0 min, before being raised to 200 °C (20 °C/min), and kept there for 5 min. The flow rates of the carrier gas (helium), make-up gas (nitrogen), hydrogen and air were 14.4, 20, 30, and 300 mL/min, respectively. For each sample, the injected volume was 1 µL and the running time was 17.5 min. The methodology has been shown to be reproducible and give good precision.

### 2.4. Lactulose/Mannitol Test in Urine

The permeability test was carried out at 9 am, as described by Meddings and Gibbons [11]. A 2-mL fresh solution containing 0.04 g/mL mannitol and 0.06 g/mL lactulose was given to each rat by oral gavage. Rats were placed individually in metabolic cages. After 2 h fasting, they were allowed free access to water. Urine was collected over 24 h into tubes attached to the cage bottom. Thymol (10% dissolved in isopropanol) was added to the collecting tubes to prevent degradation of urinary sugars due to bacterial growth. Concentrations of the sugars were measured using the EnzyChromTM Intestinal Permeability Assay Kit (EIPM-100) purchased from BioAssay System (Hayward, CA, USA).

### 2.5. Calculations and Statistical Analyses

Since the liver weights of the rats differed considerably, the pool size (total amount) of lipids may be more correct to compare. This was obtained by multiplying the analyzed concentration of lipids with the liver weight.

All statistical analyses were evaluated in GraphPad Prism software (version 7; San Diego, CA, USA). Differences in means between the high-fat control groups and the test groups were identified by one-way ANOVA and significances were then evaluated by the post-hoc Dunnett’s test. When comparing differences between only two groups (e.g., group 0 vs. group 0.5 G), an unpaired, 2-tailed *t*-test was used. Data are presented as means and their standard errors. Spearman’s test was used to find correlation between non-parametric variables. Projections to Latent Structures Discriminant Analysis (PLS-DA, SIMCA software version 15, Umetrics, Umeå, Sweden) were used to display relationships between the measured variables and the different diets. *p* < 0.05 was considered significant, while a *p* value < 0.1 was evaluated as tendency. *p* values ≤ 0.2 were reported for some variables of interest.

## 3. Results

### 3.1. Dose-Response Effects of Monobutyrin in Butter-Based Diets

#### 3.1.1. Lipids in the Liver and Portal Vein Serum

##### Liver

**Figure 1:**
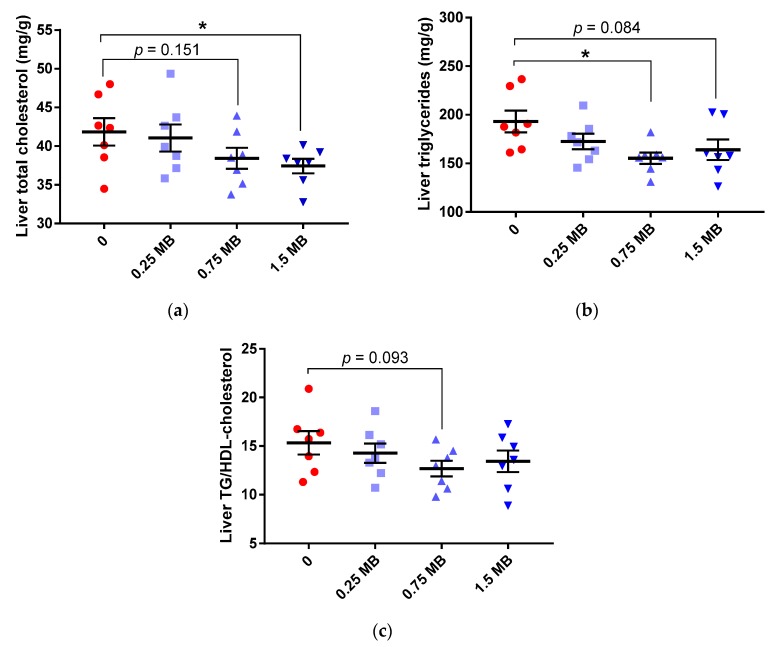
Liver lipids in rats fed a high-fat control diet with butter (0), or the same diet supplemented with 0.25 MB g/100 g (dry weight basis) (0.25 MB), 0.75 MB g/100 g (0.75 MB), or 1.5 MB g/100 g (1.5 MB) for 3 weeks. (**a**) Total cholesterol (mg/g, *p* < 0.05, unpaired, 2-tail *t*-test), (**b**) triglycerides (mg/g, *p*_ANOVA_ = 0.0447, one-way ANOVA and post-hoc Dunnett’s test), (**c**) TG/HDL-cholesterol ratio (*p* = 0.093, unpaired, 2-tail *t*-test). Values are means ± SEM. Mean values were significantly different from the control group: * *p* < 0.05. MB, monobutyrin; HDL-cholesterol, high-density lipoprotein-cholesterol; TG, triglycerides.

When increasing amounts of MB were added to a high-fat diet, there was a gradual decline in the liver concentrations of total cholesterol and triglycerides. These values reached significance for the highest amount of MB concerning cholesterol (1.5 MB group compared with the 0 group containing no MB, *p* = 0.047) and for 0.75 MB concerning triglycerides (0.75 MB group compared with the 0 group, *p* = 0.02) (Figure 1a,b, respectively). Although insignificant, the other values on total cholesterol (0.75 MB) and triglycerides (1.5 MB) were lower (*p* = 0.151 and *p* = 0.084, respectively). LDL- and HDL-cholesterol concentrations were not affected by MB and were similar between groups, but the ratio of TG-to-HDL-cholesterol, a strong factor associated with insulin resistance and cardiometabolic diseases [25], tended to be lower in rats fed the diet containing 0.75 MB than in rats consuming the 0 diet (Figure 1c, *p* = 0.093).

Glycerol 0.5 g/100 g (0.5 G) had no significant effect on liver lipids compared with the 0 group (Figure S1).

##### Portal Vein Serum

No differences were observed in portal vein serum lipids among the groups.

#### 3.1.2. Cecal Short-Chain Fatty Acids

**Figure 2:**
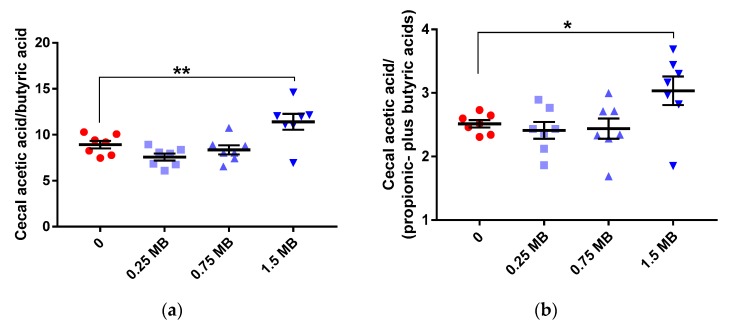
Cecal short-chain fatty acid concentrations in rats fed a high-fat control diet with butter (0), or the same diet supplemented with 0.25 MB g/100 g (dry weight basis) (0.25 MB), 0.75 MB g/100 g (0.75 MB) or 1.5 MB g/100 g (1.5 MB) for 3 weeks. (**a**) Acetic acid/butyric acid (*p*_ANOVA_ = 0.0006), (**b**) acetic acid/(propionic- plus butyric acids) (*p*_ANOVA_ = 0.0053). Values are means ± SEM. Mean values were significantly different from the control group: * *p* < 0.05, ** *p* < 0.01 (one-way ANOVA and post-hoc Dunnett’s test). MB, monobutyrin.

The cecal concentration of SCFA in rats fed the 0 diet was in total 79.7 ± 10.3 µmol/g, distributed on 46.3 ± 6.2 µmol/g for acetic acid, 13.0 ± 1.5 µmol/g for propionic acid, and 5.2 ± 0.8 µmol/g for butyric acid. When adding MB to the diet, the cecal values were very similar and no significant differences could be seen in total or individual concentrations of SCFA. However, the ratios of acetic-to-butyric acid and acetic-to-propionic- plus butyric acids were higher in the group fed 1.5 MB than the 0 group (Figure 2a,b; *p* < 0.01 and *p* < 0.05, respectively).

Addition of 0.5 glycerol g/100 g to a high-fat butter diet decreased the cecal concentration of acetic acid compared with the 0 diet in the rats (Figure S1, *p* < 0.05) and, as a tendency, also total SCFA concentrations (Figure S1, *p* = 0.082).

#### 3.1.3. Lactulose/Mannitol in Urine

**Figure 3:**
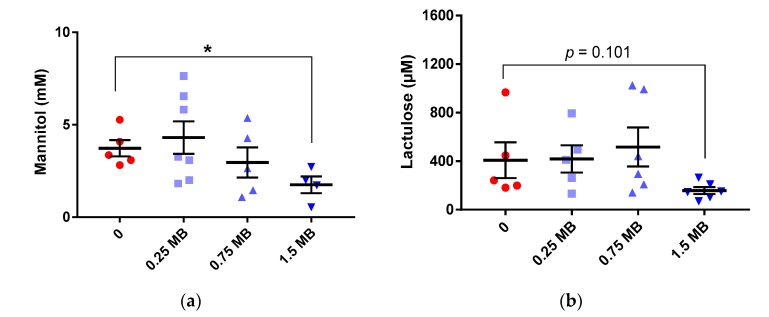
Urinary concentrations of mannitol and lactulose in rats fed a high-fat control diet with butter (0), or the same diet supplemented with 0.25 MB g/100 g (dry weight basis) (0.25 MB), 0.75 MB g/100 g (0.75 MB), or 1.5 MB g/100 g (1.5 MB) for 3 weeks. (**a**) Mannitol (mM, *p* < 0.05), (**b**) lactulose (µM). Values are means ± SEM. Mean values were significantly different from the control group: * *p* < 0.05 (unpaired, 2-tailed *t*-test). Missing data points were due to unexpectedly broken urine collecting tubes and values below detection limit. MB, monobutyrin.

There was a gradual decrease of mannitol in urine with increasing levels of MB, but the higher dose was required to give statistical significance. Thus, urinary concentration of mannitol was significantly lower in the 1.5 MB group compared with the 0 group (Figure 3a, *p* = 0.018). Furthermore, the concentration of lactulose in urine was 2.6 times lower, although insignificant (Figure 3b, *p* = 0.101 compared with the 0 group). Lactulose in urine in groups fed 0.25 MB and 0.75 MB was very similar as with the 0 group (Figure 3b). There were no significant differences in the ratio of lactulose-to-mannitol in any of the groups.

Glycerol 0.5 g/100 g had no effect on intestinal permeability compared with the 0 group (Figure S1).

#### 3.1.4. Correlations and Multivariate Data Analysis

A negative correlation was found between liver triglyceride concentrations and the cecal ratio of acetic-to-propionic- plus butyric acids (*p* = 0.074, *r* = −0.305). Furthermore, there was a negative correlation between LDL-cholesterol concentrations and cecal concentrations of total SCFA (*p* = 0.042, *r* = −0.346), acetic acid (*p* = 0.007, *r* = −0.447), propionic acid (*p* = 0.042, *r* = −0.041), and butyric acid (*p* = 0.059, *r* = −0.322).

Lactulose in urine correlated negatively with cecal concentrations of acetic acid (*r* = −0.326, *p* = 0.091), and the ratio of lactulose and mannitol with total SCFA (*r* = −0.374, *p* = 0.042), acetic acid (*r* = −0.492, *p* = 0.006), propionic acid (*r* = −0.344, *p* = 0.063) and acetic-to-isobutyric- plus isovaleric acids (*r* = −0.401, *p* = 0.028).

**Figure 4:**
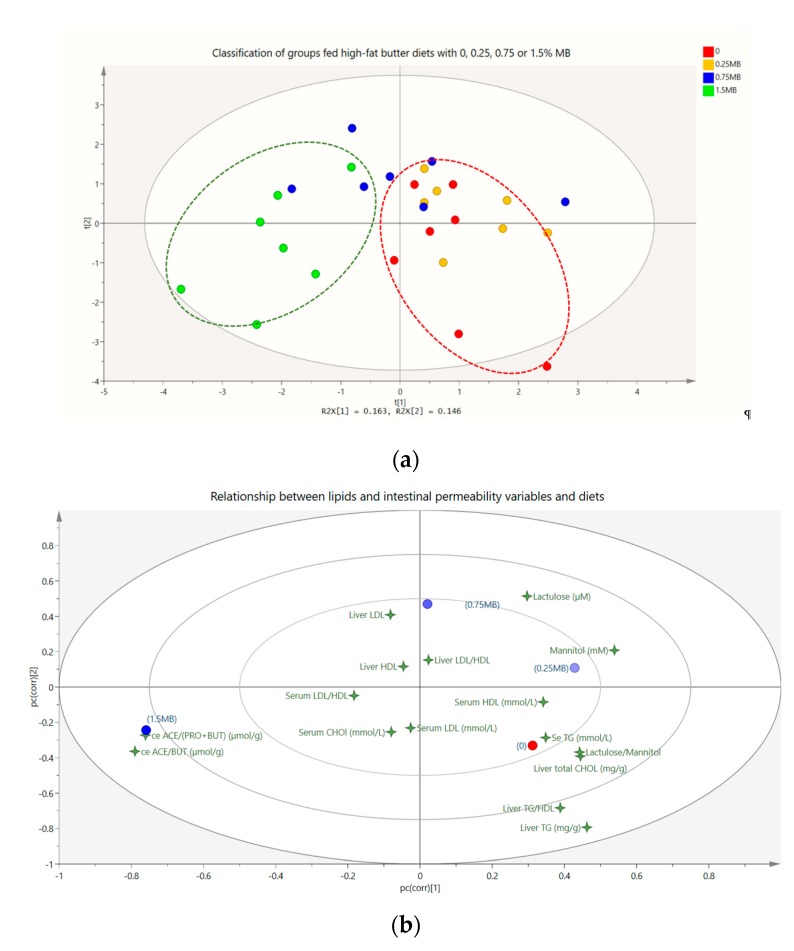
Lipid profiles and intestinal permeability in rats fed a high-fat control diet with butter (0), or the same diet supplemented with 0.25 MB g/100 g (dry weight basis) (0.25 MB), 0.75 MB g/100 g (0.75 MB), or 1.5 MB g/100 g (1.5 MB) for 3 weeks. (**a**) Score scatter plot shows separation of the treatment groups, especially 1.5 MB (green dashed line) compared to high-fat control diet with butter (0) (red dashed line), (**b**) loading scatter plot displays variables (4-point stars) of lipids and intestinal permeability in relation to the treatment groups (circles). Variables responsible for separation of groups fed the 0 and 1.5 MB diets are lactulose, mannitol, lactulose/mannitol ratio, liver total cholesterol, liver triglycerides, liver triglycerides/HDL-cholesterol ratio, cecal acetic acid-to-butyric acid ratio, and cecal acetic acid-to-propionic- plus butyric acids ratio. MB, monobutyrin; ACE, acetic acid; PRO, propionic acid; BUT, butyric acid; LDL, low-density lipoprotein-cholesterol; HDL, high- density lipoprotein-cholesterol; CHOL, cholesterol; TG, triglycerides.

A summary of results on lipids and intestinal permeability is shown in Figure 4. Analysis of results in the PLS-DA model shows that the first component (horizontal axis) is significantly different when cross validated, which means that there is a separation between groups. In Figure 4a, each dot represents all results (lipids and mannitol/lactulose) for one rat. Interestingly, all rats in group 1.5 MB, and to some extent also those in the group 0.75 MB, are in the opposite direction to rats in group 0 and 0.25 MB. Thus, increasing doses of MB (on the left side at the horizontal axis) increase the difference between the treatment groups and the 0 group (marked in red dashed line), with the clearest separation found for 1.5 MB (marked in green dashed line). The corresponding loading plot (Figure 4b) shows which variables that are responsible for the variation between groups. Assisted with Variable Importance for the Projection (VIP) analysis, variables that have discriminating power are lactulose, mannitol, lactulose/mannitol ratio, liver total cholesterol, liver triglycerides, liver triglycerides/HDL-cholesterol ratio, cecal acetic acid-to-butyric acid ratio, cecal acetic acid-to-propionic- plus butyric acids ratio. The smaller the distance between a specific variable and a group, the closer the connection. In other words, the 0 group has increased values of total cholesterol, triglycerides and TG/HDL-cholesterol ratio in the liver as well as increased lactulose/mannitol ratio in the urine, but decreased ratios of acetic acid-to-butyric acid and of acetic acid-to-propionic- plus butyric acids (Figure 4b). These variables are reversed with the 1.5 MB group, which results are located on the opposite side of the 0 group.

### 3.2. Effects of Monobutyrin in Lard-Based Diets

#### 3.2.1. Lipids in the Liver Tissue and Serum from Portal Vein, Aorta and Tail

##### Liver

**Figure 5:**
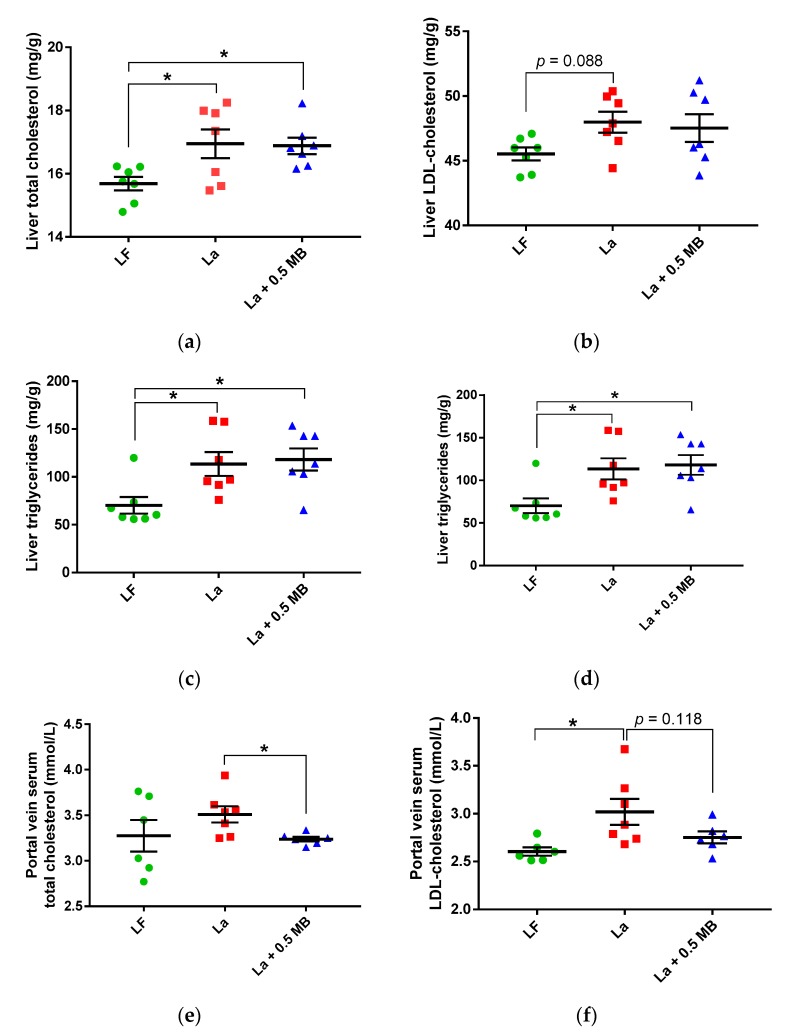
Lipid profiles in rats fed a low-fat (LF) diet, a high-fat control diet based on lard (La), or the same La diet supplemented with 0.5 MB g/100 g (dry weight basis) (La + 0.5 MB) for 4 weeks. (**a**) Liver total cholesterol (mg/g, *p*_ANOVA_ = 0.0116, one-way ANOVA and post-hoc Dunnett’s test), (**b**) liver LDL-cholesterol (mg/g, *p*_ANOVA_ = 0.1092, one-way ANOVA and post-hoc Dunnett’s test), (**c**) liver triglycerides (mg/g, *p*_ANOVA_ = 0.0116, one-way ANOVA and post-hoc Dunnett’s test), (**d**) total amount of liver HDL-cholesterol (mg, *p*_ANOVA_ = 0.04, one-way ANOVA and post-hoc Dunnett’s test) (**e**) portal vein serum total cholesterol (mmol/L, *p* < 0.05, unpaired, 2-tailed *t*-test), (**f**) portal vein serum LDL-cholesterol (mmol/L, *p* < 0.05, unpaired, 2-tailed *t*-test). Values are means ± SEM. Mean values were significantly different between groups: * *p* < 0.05. LDL-cholesterol, low-density lipoprotein-cholesterol; HDL-cholesterol, high-density lipoprotein-cholesterol.

The concentrations of total cholesterol (Figure 5a, *p* < 0.05), LDL-cholesterol (Figure 5b, *p* = 0.088) and triglycerides (Figure 5c, *p* < 0.05) were higher in the group fed the high-fat La control diet than the group fed the LF diet. Addition of 0.5 MB g/100 g did not change these results. However, the total amount of liver HDL-cholesterol (i.e., concentration multiplied with liver weight) was significantly higher in the La + 0.5 MB group compared with the control group (Figure 5d, *p* < 0.05).

##### Portal Vein Serum

Rats fed the La + 0.5 MB diet had significantly lower portal vein serum concentrations of cholesterol (Figure 5e, *p* = 0.02) and lower LDL-cholesterol concentrations (insignificant, Figure 5f, *p* = 0.118) compared with rats fed the La control diet. The values with La + 0.5 MB were very similar as with the LF diet. No difference could be seen in portal vein concentrations of HDL-cholesterol and triglycerides between any of the groups (Figure S2a,b).

##### Tail and Aortic Serum

Mean cholesterol concentrations in the rat tail venous blood decreased considerably during the intervention period with the LF diet, while La and La + 0.5 MB were quite similar. This resulted in significantly lower cholesterol values in the LF group compared with the La and La + 0.5 MB groups after week 3 (Figure S2c, *p* < 0.05). Although no differences were found in mean cholesterol concentrations for the whole groups, individual analyses on each rat were shown to decrease significantly after 3 weeks for four of the rats fed La + 0.5 MB diet (from 4.13 to 3.89 mmol/L, *p* = 0.036) and for six rats fed the LF diet (from 4.02 to 3.25 mmol/L, *p* = 0.0075). In the group fed La + 0.5 MB, one rat had similar cholesterol levels during the intervention, while two increased their levels. 

Concerning the ratio LDL-cholesterol/HDL-cholesterol, this was lower already after week 1 for the La + 0.5 MB group compared with the La group (Figure S2d, *p* < 0.05). However, after week 3 there was a considerable decrease in this ratio for all diets, resulting in similar values. No differences could be seen in other lipids in blood collected from the tail vein.

The different lipid concentrations were very similar in the aortic blood. However, the ratio of TG/HDL-cholesterol tended to be lower in the LF and La + 0.5 MB groups than in the La group (Figure S2e, *p* = 0.105 and *p* = 0.122, respectively).

#### 3.2.2. Correlation and Multivariate Data Analysis

Spearman correlation test showed a positive relationship between portal vein LDL-cholesterol with portal vein TG (*p* = 0.031, *r* = 0.495) and liver LDL-cholesterol (*p* = 0.013, *r* = 0.556) and TG (*p* = 0.002, *r* = 0.674).

Effects of MB in lard-based diets are shown in Figure S3 in a PLS-DA plot. Addition of 0.5 MB g/100 g to a lard diet shifts the location of the La + 0.5 MB group to the opposite side of the La group, i.e., to the same side where values from the LF group are located (Figure S3a). In line with this, higher values of LDL-cholesterol and LDL-cholesterol/HDL-cholesterol in the portal vein and TG/HDL-cholesterol in the aorta were associated with the La group, while these variables were lower in the La + 0.5 MB and LF groups (Figure S3b).

### 3.3. Effects of Monobutyrin on Body-Weight Gain and Organ Weights

#### 3.3.1. Butter Diets

Groups fed the MB-supplemented diets did not differ from the 0 group in final body weight and tissue weights, except for the 1.5 MB group that tended to have higher relative liver weights compared with the 0 group (Table S2, *p* = 0.087).

Glycerol in the diet (0.5 G) increased the final body weight and body weight gain (*p* = 0.019 and *p* = 0.013, respectively) compared with the 0 group, and tended to give heavier liver weights (*p* = 0.057).

#### 3.3.2. Lard Diets

Rats given the high-fat diets consumed less amount of food but gained more weight than those on the LF diet, as shown by the higher food efficiency ratio (body weight gain/total food intake) for the La and La + 0.5 MB groups (Table S2, *p* < 0.05). Relative weights of liver and spleen were significantly lower in the La group compared with the LF group (*p* < 0.05). No differences concerning organ weights could be seen between the La and La + 0.5 MB group.

## 4. Discussion

### 4.1. Dose-Dependent Lowering Effect on Liver Total Cholesterol

Addition of MB to a high-fat butter diet decreased diet-induced elevated liver total cholesterol concentrations in a dose-dependent manner. This result is consistent with our previous work where a similar reduction in liver cholesterol was accompanied by down-regulation of genes involved in bile acid synthesis (specifically, cholesterol 7-alpha-hydroxylase/*Cyp7a1* and sterol 12-alpha-hydroxyalse/*Cyp8b1*) in rats fed high-fat diets supplemented with 0.5 MB g/100 g [17]. However, in that study we also observed a decrease in liver LDL-cholesterol concentrations, which could not be seen in the present study. The reason for these discrepancies is currently unknown. It may be speculated that MB is acting preferably on cholesterol absorption/excretion via bile acid metabolism rather than directly on LDL-cholesterol uptake by the liver, although there are also other factors that can influence cholesterol metabolism. Indeed, SCFA have shown to reduce hepatic cholesterol levels by lowering substrates for cholesterol synthesis (e.g., liver 3-hydroxy-3-methylglutaryl-CoA), suppressing genes regulating these substrates (e.g., ATP-citrate lyase), or promoting fecal bile acid excretion [14,15]. Specifically, butyric acid has been shown to influence intestinal lipid absorption by inhibiting expression of microsomal triglyceride transfer protein [16].

It should be mentioned that the total cholesterol measured in this study included both esterified and free cholesterol, originally present in samples. Free/unesterified cholesterol seems to have more negative effects than esterified cholesterol under certain diseased conditions. For instance, faster removal from blood followed by greater vessel tissue deposition of free cholesterol compared with cholesteryl ester was found in coronary artery disease (CAD) patients [26]. Furthermore, diminished transfer of unesterified cholesterol to HDL was reported in CAD patients with and without type 2 diabetes [27]. However, it should be mentioned that cholesterol transfer may differ between humans and rats, since rats are naturally deficient in cholesteryl ester transfer protein. Recent data in CAD patients indicated the ratio of unesterified/esterified cholesterol in lipoprotein fractions as a stronger predictor of atherogenicity than other cholesterol subfractions [28]. Thus, further investigations of the presence of different lipids may reveal potential impact of MB supplementation on cholesterol esterification profile.

### 4.2. Reduction of Liver Triglycerides

Apart from the observed lowering effect of MB on total liver cholesterol, reduction in liver triglyceride concentrations was also found in the groups fed the MB diets in a dose-response manner, with effects seen already at 0.75 MB g/100 g. No effects on triglycerides could be seen in our previous study where a dose of 0.5 MB g/100 g was added to butter-based diets [17]. The reason for these diverging results is not strange per se, and may be attributed to the lower dose used in that study. Thus, MB inhibits glycerol uptake in the rat, leaving less substrate available for the formation of triglyceride backbone [29]. SCFA are also able to decrease liver triglycerides by promoting lipid oxidation, and to suppress lipolysis and non-esterified fatty acids in blood via activation of their receptors such as GPR109A and GPR43 (highest affinity to acetate) [30,31,32]. Furthermore, acetic acid is reported to suppress triglyceride biosynthesis by upregulating expression of acyl-CoA oxidase, an enzyme involved in fatty acid oxidation [15]. Although there were no changes in acetic acid concentration in the present study, higher ratios of acetic-to-propionic- plus butyric acids were seen in rats fed the 1.5 MB diet compared with those in the control group, suggesting a potential reverse relationship between acetic acid and liver triglycerides.

Although indirect, it can be of importance to discuss the reduced liver triglycerides in association with activation of peroxisome proliferator-activated receptor alpha (PPARα), a ligand-activated transcription factor involved in regulation of genes that stimulate removal of triglycerides from the bloodstream and enhance β-oxidation in the liver. Tributyrin, a similar sibling of monobutyrin, has been shown to increase mRNA and protein expression of PPARα, and β-oxidation related genes in rat liver [33]. These effects were mediated by increased acetylation of histone H3 in the liver, subsequently leading to inhibition of inflammatory markers and liver injury, and reduced levels of plasma triglycerides, total and LDL-cholesterol after 24 h LPS challenge. Especially, fibrates, ligands of PPARα are clinically used to treat dyslipidemia [34]. In addition, activation of PPARα also reduced hepatic expression of a glycerol transporter aquaporin 9 (AQP9) in male Wistar rats [35]. This evidence suggests the effects of MB could be explained by similar mechanisms. However, further investigations with specific aims are needed to confirm these potential suggestions.

Positive effects of MB as an effective hepatic glycerol uptake inhibitor have been reported [29]. Our current data show that supplementation of MB to the high-fat diets caused reduction in liver concentrations of cholesterol and triglycerides, further establishing a potent impact of MB on lipid metabolism. Indeed, dietary supplementation of MB given to rats on high-fat diets led to a reduction in liver succinic acid [17], a metabolite elevated in metabolic diseases, such as diabetes, and capable of increasing blood pressure via activation of its receptor GPR91 [36,37]. Furthermore, tributyrin has been shown to reduce liver triglyceride accumulation and insulin levels in high-fat fed mice [38]. Tributyrin lipid emulsion was reported to bind to LDL and selectively taken up by cellular LDL receptors [39]. Therefore, it remains to be determined if there is a link between the effects of MB and these receptors or other SCFA receptors.

### 4.3. Improved Intestinal Barrier Function

Consumption of high-fat diets compromises the gut barrier both in rodents and humans, facilitating the onset of obesity and associated metabolic disorders [4,40,41]. Studies in rats have shown that there is a considerable increase in paracellular permeability seen as early as within one week of high-fat feeding in the small intestine, and after 3 weeks in the colon, coinciding with elevated LPS binding protein (LBP) in blood [2]. Studies have failed to show that MB in dose of 0.5 g/100 g in the high-fat diet can lower blood LBP levels in rats [17] while tributyrin in dose 2.0 g/kg body weight can change LPS levels in mice [38]. However, sustained expression and co-localizations of tight junction proteins, and reduced intestinal permeability were reported in mouse models of gut injury or colitis after tributyrin treatments [9,42]. These data led us to the hypothesis that MB could have potential effects on intestinal paracellular permeability. Intriguingly, the present data showed a prominent reduction in urinary concentrations of mannitol, as well as a decreased ratio of lactulose to mannitol (although insignificant) in the rats fed the high-fat diet supplemented with 1.5 MB g/100 g. The urinary excretion of these two sugars has been evaluated as a good indicator of small intestinal in vivo permeability in rats [11]. In humans, increased ratio of lactulose-to-mannitol in urine has been found in diabetes and obesity [12,41,43]. In fact, increase in this ratio is also linked to an increase in insulin and a decline in HDL-cholesterol concentration in obese subjects [41]. The decreasing effect of MB on intestinal permeability found in this study may provide a probable mechanism linking obesity-related symptoms and gut permeability as previously seen in humans [12,41,43]. Altogether, the data from this study show, for the first time that MB, at a dose of 1.5 g/100 g, is protective against high-fat diet-induced gut impairment by partly modulating small intestinal barrier function, complementing the anti-inflammatory actions of MB previously reported [17].

Another compound that needs to be considered in relation to gut permeability is glycerol, which accounted for 39% (*w/w*) of MB composition. The results from the dose-response study did not show any differences in lipid profiles and intestinal permeability between the glycerol-fed group and the high-fat control group, except a significant decrease in cecal concentrations of acetic acid in the group fed glycerol. Therefore, the effect of glycerol seems less probable and the observed lipid-lowering impact of MB was solely linked to MB.

### 4.4. Effect of Fat Source on Lipid Profiles

It may be discussed why the effects of MB could be seen on portal vein cholesterol and LDL-cholesterol in rats fed lard-based diets, but not in rats receiving butter-based diets. One reason could be the difference in lipid composition between butter and lard. Thus, the cholesterol content in butter is higher than in lard (219 vs. 95 mg/100 g, respectively) [21,44] which makes it harder to affect cholesterol results. It cannot be excluded that the longer experimental time in the lard-based diet study (4 weeks) than in the butter-based diet study (3 weeks) was another reason for the discrepancies. Furthermore, butter has a higher ratio of saturated-to-polyunsaturated acids compared to lard (16.8 vs. 3.5). Saturated fatty acids, especially palmitic acid, was shown to reduce LDL-receptors in the liver, resulting in a subsequent elevation of LDL in blood [45]. However, the distribution of saturated fatty acids at the stereospecific numbering position 2 (sn-2) of triglycerides is higher in lard than in butter (59 vs. 39 mol %), and saturated fatty acids attached at this position are preferentially delivered to the liver [46], potentially explaining the lowering effects of MB on liver lipids seen in rats fed butter-diets, but not with those fed lard-diets. Besides these dietary factors, it should be emphasized that blood cholesterol is regulated by many factors, such as intestinal cholesterol absorption, hepatic cholesterol synthesis, as well as biliary excretion and cellular use [47]. Of note, SCFA, including butyric acid, exert a wide impact on these factors as mentioned throughout the paper.

Saturated fat is an important factor when inducing metabolic disorders in animal models, such as increased levels of total cholesterol, LDL-cholesterol, triglycerides, and decreased HDL-cholesterol [48]. Lard is generally used as the fat source in both mice and rat studies. However, lard is not that commonly used in human diets, while butter is, and this was also the reason why we used butter initially. Both fat sources have high amounts of saturated fatty acids, although the composition is even poorer in butter than in lard. On the other hand, butter has been reported to contain 3–4 g/100 g of tributyrin [21] and 0.189 mM/100 g of extracted fat of monoglycerides [20], which might affect/change the results in a positive way. In this respect, it is important to point out that the presence of MB and TB in butter used in our study was below detection of limit (<0.001% weight) by using gas-chromatography. With this background, we decided to evaluate effects of MB on lipid profile when lard was used as the main fat source. As expected, presence of lard strongly elevated lipids in the liver, while adding of MB in the lard diet tended to increase liver HDL-cholesterol concentrations, also leading to a consequent decline in LDL-cholesterol/HDL-cholesterol ratio. Furthermore, and perhaps more importantly, the concentrations of total cholesterol in portal vein blood were lower (*p* = 0.02) in the MB-fed rats than in rats fed high-fat control containing lard. The same changes could also be seen in LDL-cholesterol concentrations that remained similar between the LF and MB group. These results could be possibly linked to the ability of SCFA to inhibit lipolysis and plasma free fatty acids via activation of GPR43 or GPR109A [31]. Improvements such as HDL elevation in treating dyslipidemia have also been reported for GPR109A [49]. Overall, the lipid lowering effects of MB are persistent, with parameters of lipid profile being differently affected depending on the organs/locations and fat source investigated.

**Figure 6:**
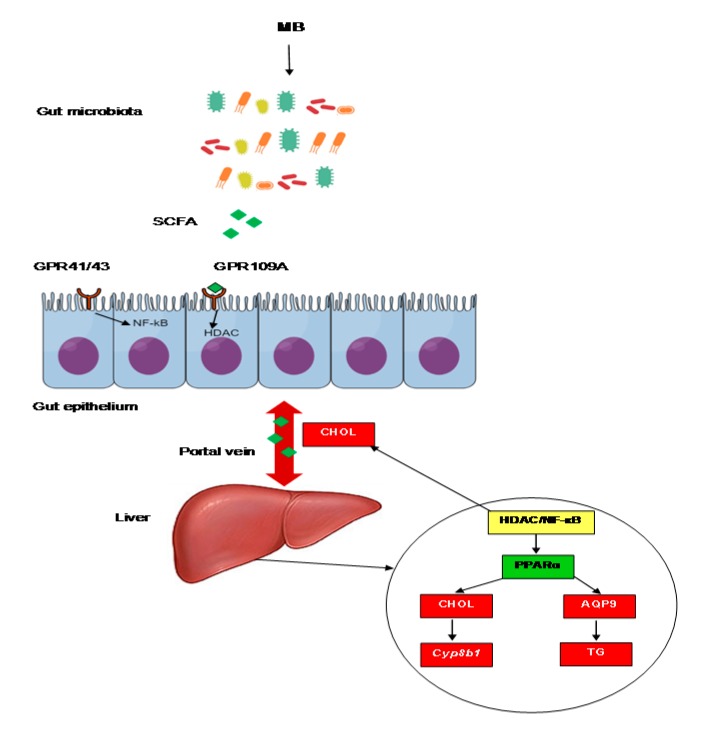
Possible mechanisms involved in effects of monobutyrin (MB) on lipid profile and intestinal permeability. MB is hydrolysed to butyric acid (BUT), altering cecal microbiota composition. When transported to colonic cells via receptors (GPR41/43 or GPR109A), BUT decreases intestinal permeability by enhancing expression of tight junction proteins and suppresses intestinal inflammation and lipid absorption via inhibition of HDAC or NF-κB. Via the portal vein, BUT can reach the liver where inhibition of HDAC/NF-κB by BUT leads to reduction in cholesterol and triglycerides/glycerol transporter APQ9 through PPARα activation. Decreased hepatic cholesterol results in downregulation of bile acid related gene *Cyp8b1*. Changes in liver lipids eventually cause decreased lipids in serum and the intestine. SCFA, short-chain fatty acids; GPR, G-coupled protein receptors; NF-κB, nuclear factor-kappa B; HDAC, histone deacetylase; CHOL, cholesterol; *Cyp8b1*, sterol 12-alpha-hydroxylase; PPARα, peroxisome proliferator-activated receptor alpha; AQP9, aquaporin 9; TG, triglycerides.

Finally, it could be questioned whether the effects seen in this study are coming from MB as a free compound or its metabolites. A complete answer cannot be achieved with the design and aim of this study since it is not known whether the enzymatic hydrolysis of MB to butyric acid is complete or not. However, taking into account reported positive effects of MB and tributyrin on cecal microbiota composition, liver lipids, high-fat diet-induced inflammation, and gut barrier function [9,17,33,38], it can be suggested that mechanisms responsible for the effects of MB are possibly related to main functions of butyric acid, i.e., inhibition of histone deacetylase and regulation of receptor-mediated signaling. A summary of effects and mechanisms of MB is illustrated in Figure 6. Future studies using methodologies that can differentiate effects of MB and its metabolites in the body may help to clarify this matter.

## 5. Conclusions

In conclusion, the present study confirms the liver cholesterol-lowering effect of MB in a dose-responding manner with the most considerable effect seen for 1.5 MB g/100 g diet. Moreover, intestinal barrier function was significantly improved with this dose of MB. Generally, MB supplementation was effective in lowering portal vein blood cholesterol when mixed into high-fat diets with lard as a fat source. These findings suggest promising potential of MB as a dietary supplement to prevent metabolic disturbances and reduce the risk factors for disease development associated with high-fat consumption.

## Supplementary Materials

The following figures are available online at http://www.mdpi.com/2072-6643/11/2/308/s1: Figure S1. Effects of glycerol on cecal SCFA, liver lipids and intestinal permeability in rats fed a high-fat control diet with butter (0) or the same diet supplemented with 0.5 glycerol g/100 g (dry weight basis) (0.5 G) for 3 weeks; Figure S2. Serum lipids in rats fed a low-fat (LF) diet, a high-fat control diet based on lard (La), or the same La diet supplemented with 0.5 MB g/100 g (dry weight basis) (La + 0.5 MB) for 4 weeks; Figure S3. Effects of MB on lipids profiles in rats fed a low-fat (LF) diet, a high-fat control diet with lard (La), or the same diet supplemented with 0.5 MB g/100 g (dry weight basis) (La + 0.5 MB) for 4 weeks; Table S1. Composition of the experimental diets (g/kg dry weight); Table S2. Final body weight (g), body weight gain (g), actual and relative (normalized to body weight) tissue weights (g and % body weight), total food intake (mean g/rat), food efficiency ratio (body weight gain/food intake, g/g) in rats fed a high-fat control diet with butter (0), or the same diet supplemented with 0.25 MB g/100 g (dry weight basis) (0.25 MB), 0.75 MB g/100 g (0.75 MB), 1.5 MB g/100 g (1.5 MB), or 0.5 glycerol g/100 g (0.5 G) for 3 weeks, or in rats fed a low-fat (LF) diet, a high-fat control diet with lard (La), or the same diet supplemented with 0.5 MB g/100 g (La + 0.5 MB) for 4 weeks. MB, monobutyrin.
