# Correction: Nguyen et al. “Monobutyrin Reduces Liver Cholesterol and Improves Intestinal Barrier Function in Rats Fed High-Fat Diets” *Nutrients* 2019, *11*, 308

**DOI:** 10.3390/nu11092106

**Published:** 2019-09-04

**Authors:** Thao Duy Nguyen, Olena Prykhodko, Frida F. Hållenius, Margareta Nyman

**Affiliations:** Department of Food Technology, Engineering and Nutrition, Kemicentrum, Lund University, P.O. Box 124, SE-221 00 Lund, Sweden (O.P.) (F.F.H.) (M.N.)

## Excerpt

The authors wish to make a correction to the published version of their paper [1].

Figure 5d in the published paper is not correct One graph “(c) Liver triglycerides (mg/g)” was accidentally duplicated where graph “(d). Total amount of liver HDL-cholesterol (mg)” should have been. A correct version of the figure is included below.

The authors apologize to the readers for any inconvenience caused by the change. This change does not impact on the text of the paper or on the overall results or scientific conclusions. The original manuscript will remain online on the article webpage, with a reference to this correction.

## Figures and Tables

**Figure 5:**
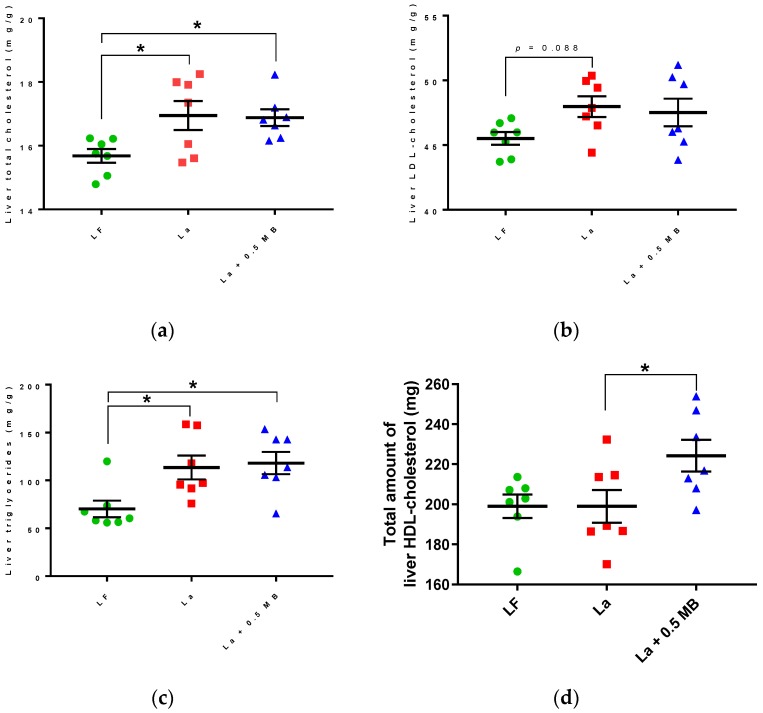

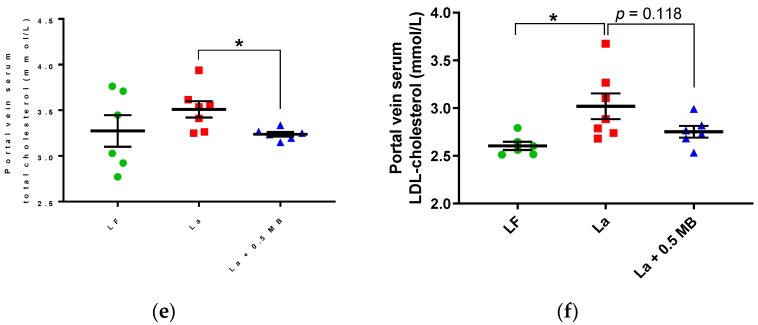
Lipid profiles in rats fed a low-fat (LF) diet, a high-fat control diet based on lard (La), or the same La diet supplemented with 0.5 MB g/100 g (dry weight basis) (La + 0.5 MB) for 4 weeks. (**a**) Liver total cholesterol (mg/g, *p*_ANOVA_ = 0.0116, one-way ANOVA and post-hoc Dunnett’s test); (**b**) liver LDL-cholesterol (mg/g, *p*_ANOVA_ = 0.1092, one-way ANOVA and post-hoc Dunnett’s test); (**c**) liver triglycerides (mg/g, *p*_ANOVA_ = 0.0116, one-way ANOVA and post-hoc Dunnett’s test); (**d**) total amount of liver HDL-cholesterol (mg, *p*_ANOVA_ = 0.04, one-way ANOVA and post-hoc Dunnett’s test); (**e**) portal vein serum total cholesterol (mmol/L, *p* < 0.05, unpaired, two-tailed *t*-test); (**f**) portal vein serum LDL-cholesterol (mmol/L, *p* < 0.05, unpaired, two-tailed *t*-test). Values are means ± SEM (standard error of the means). Mean values were significantly different between groups: * *p* < 0.05. LDL-cholesterol, low-density lipoprotein-cholesterol; HDL-cholesterol, high-density lipoprotein-cholesterol.
