# Supplementary material for: Monobutyrin Reduces Liver Cholesterol and Improves Intestinal Barrier Function in Rats Fed High-Fat Diets
Source: Nutrients. 2019 Feb 1;11(2):308. doi: 10.3390/nu11020308 (PMC6412756; doi:10.3390/nu11020308)
Supplement: Supplementary file 1 [file nutrients-11-00308-s001.pdf]

Table 1. Composition of the experimental diets (g/kg dry weight)

Components/Diets	0	0.25 MB	0.75 MB	1.5 MB	0.5 G	LF	La	La + 0.5 MB
Casein†	150.0	150.0	150.0	150.0	150.0	150.0	150.0	150.0
DL-Methionine†	1.2	1.2	1.2	1.2	1.2	1.2	1.2	1.2
Butter‡	180.0	180.0	180.0	180.0	180.0	-	-	-
Rapeseed oil	50.0	50.0	50.0	50.0	50.0	50.0	-	-
Sucrose	100.0	100.0	100.0	100.0	100.0	100.0	100.0	100.0
Cellulose§	50.0	50.0	50.0	50.0	50.0	50.0	50.0	50.0
Mineral mixture‖	48.0	48.0	48.0	48.0	48.0	48.0	48.0	48.0
Vitamin mixture‖	8.0	8.0	8.0	8.0	8.0	8.0	8.0	8.0
Choline chloride†	2.0	2.0	2.0	2.0	2.0	2.0	2.0	2.0
Monobutyrin	0.0	2.5	7.5	15.0	-	-	-	5.0
Glycerol	-	-	-	-	5.0	-	-	-
Lard	-	-	-	-	-	-	230	230
Wheat starch¶	410.8	408.3	403.3	395.8	405.8	590.8	410.8	405.8

MB, monobutyrin; G, glycerol; LF, low-fat; La, lard; † Sigma-Aldrich, St. Louis, MO, USA; ‡ Arla Foods, Stockholm, Sweden; § FMC BioPolymers, Cork, Ireland; ‖ Altromin, Lage, Germany; Cargill, Sas van Gent, The Netherlands; varied depending on the ester and fat content of the test diets.

Table 2. Final body weight (g), body weight gain (g), actual and relative (normalized to body weight) tissue weights (g and % body weight), total food intake (mean g/rat), food efficiency ratio (body weight gain/food intake, g/g) in rats fed a high-fat control diet with butter (0), or the same diet supplemented with 0.25 MB g/100g (dry weight basis) (0.25MB), 0.75 MB g/100g (0.75MB), 1.5 MB g/100g (1.5MB), or 0.5 glycerol g/100g (0.5G) for 3 weeks, or in rats fed a low-fat (LF) diet, a high-fat control diet with lard (La), or the same diet supplemented with 0.5 MB g/100g (La + 0.5 MB) for 4 weeks. MB, monobutyrin.

Butter-based diets (3-week study)						
**Variables/Groups**	**0**	**0.25 MB**	**0.75 MB**	**1.5 MB**	**0.5 G**	***p* ANOVA**
Final body weight	277 ± 9.9	274 ±7.4	284 ± 8.5	295 ± 8.0	317 ± 10.6*	0.0128
Body weight gain	162.5 ± 9.0	159.6 ± 6.1	165.9 ± 5.7	180.9 ± 8.3	195.1 ± 8.6*	0.0132
Total food intake	366.7	403.9	421.3	441.9	421.4	-
FER	0.44 ± 0.02	0.40 ± 0.02	0.39 ± 0.01	0.41 ± 0.02	0.46 ± 0.02	ns
Liver weight (g)	11.1 ± 0.7	11.6 ± 0.3	10.8 ± 0.5	12.8 ± 0.6	13.1 ± 0.7	ns
Liver weight (%)	4.0 ± 0.1	4.3 ± 0.1	3.8 ± 0.1	4.3 ± 0.1	4.1 ± 0.1	ns

Lard-based diets (4-week study)				
**Variables/Groups**	**LF**	**La**	**La + 0.5 MB**	***p* ANOVA**
Final body weight	345 ± 7.4	363 ± 8.1	384 ± 7.2##	0.0074
Body weight gain	152.6 ± 4.2	160.6 ± 4.7	169.2 ± 4.8#	0.0209
Total food intake	661.9	601.8	599.4	-
FER	0.23 ± 0.01	0.27 ± 0.01#	0.28 ± 0.01###	0.0004
Liver weight (g)	15.1 ± 0.6	14.1 ± 0.3	15.3 ± 0.4	ns
Liver weight (%)	4.4 ± 0.1	3.9 ± 0.1##	4.0 ± 0.1#	0.0036
Spleen weight (%)	0.26 ± 0.02	0.22 ± 0.01#	0.25 ± 0.01	0.0452
Brain weight (%)	0.57 ± 0.01	0.54 ± 0.01	0.52 ± 0.01#	0.0138
Abdominal fat (%)	2.4 ± 0.21	3.1 ± 0.28	3.2 ± 0.14#	0.0274
Epididymal fat (%)	1.4 ± 0.1	1.8 ± 0.1	1.8 ± 0.1#	0.0180
SI length (cm)	81.7 ± 1.3	87.1 ± 2.0	88.7 ± 1.0#	0.0107

FER, food efficiency ratio; SI, small intestine; "-", not applicable; ns, not significant; Values are mean ± SEM, $n = 7$; Mean values were significantly different from values of control group with butter: * $p < 0.05$ (one-way ANOVA and post-hoc Dunnett's test); Mean values were significantly different from values of the LF group: # $p < 0.05$, ## $p < 0.01$, ### $p < 0.001$ (one-way ANOVA and post-hoc Dunnett's test).

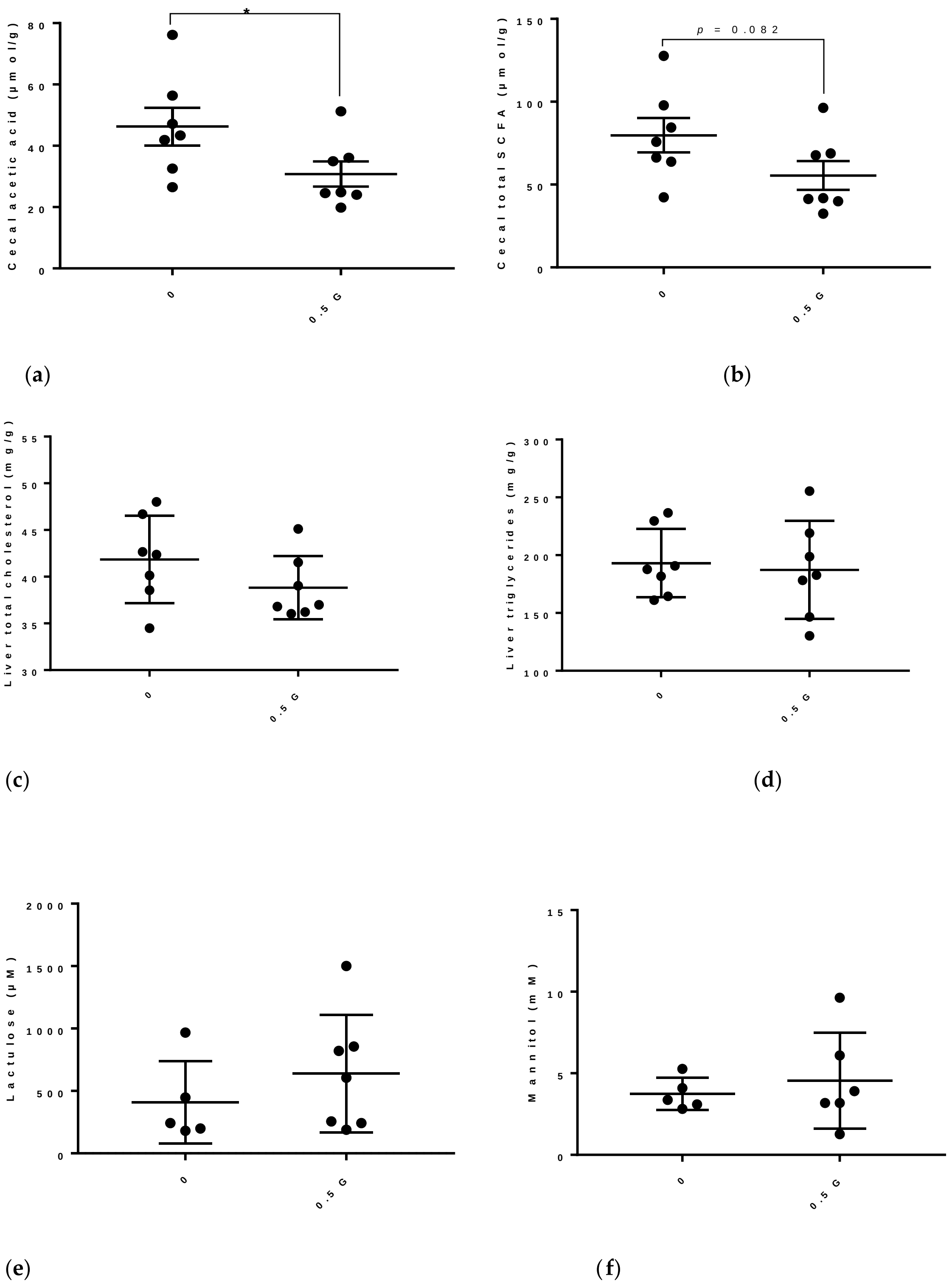

Figure 1. Effects of glycerol on cecal short-chain fatty acids, liver lipids and intestinal permeability in rats fed a high-fat control diet with butter (0) or the same diet supplemented with 0.5 glycerol g/100 g (0.5 G) for 3 weeks. (**a**) Acetic acid (μmol/g), (**b**) total SCFA (μmol/g), (**c**) total cholesterol (mg/g), (**d**) triglycerides (mg/g), (**e**) lactulose (μM), (**f**) mannitol (mM). Values are means ± SEM. Mean values were significantly different from the control group: * $p < 0.05$. SCFA, short-chain fatty acids.

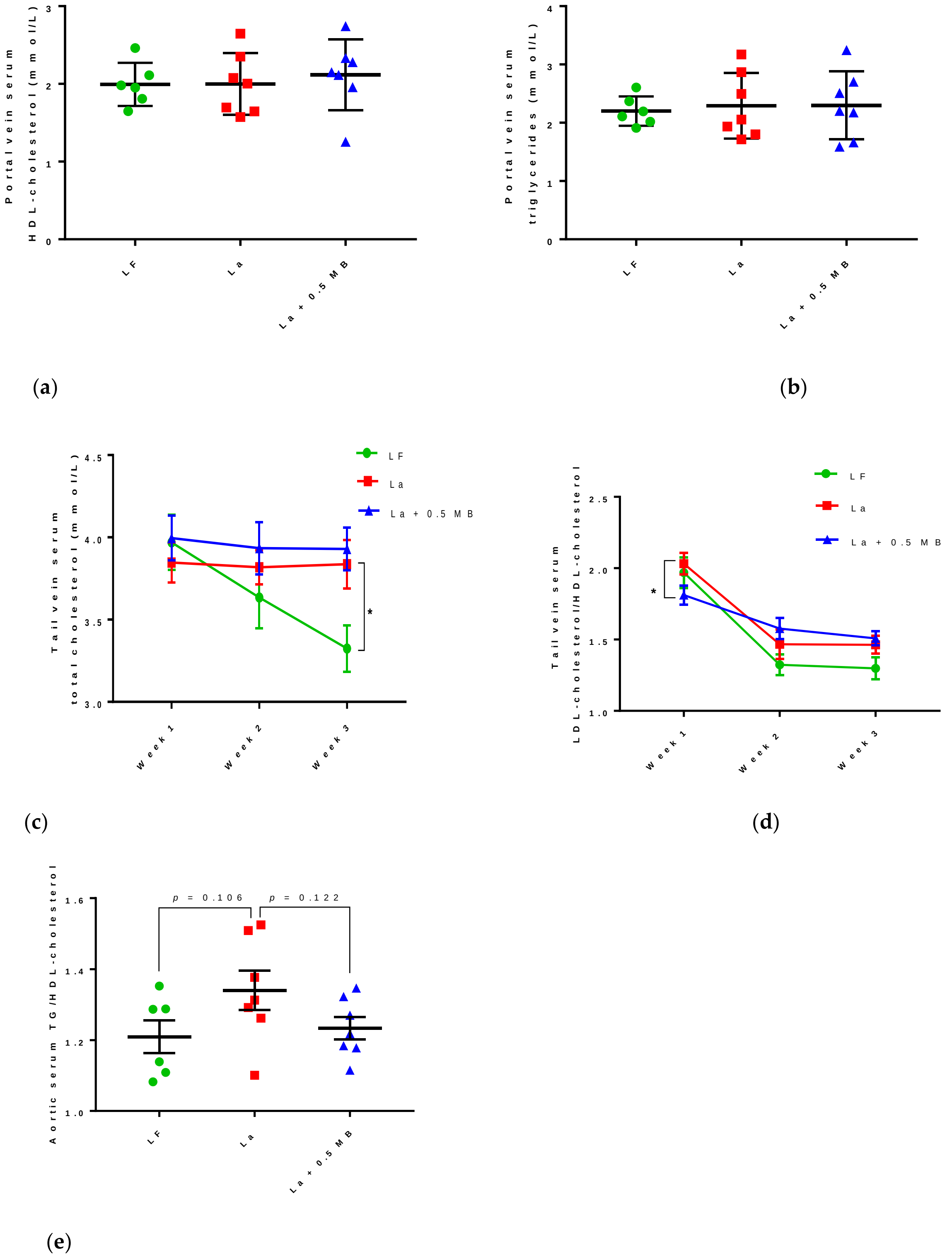

Figure 2. Serum lipids in rats fed a low-fat (LF) diet, a high-fat control diet based on lard (La), or the same La diet supplemented with 0.5 MB g/100g (dry weight basis) (La + 0.5 MB) for 4 weeks. (**a**) Portal vein serum HDL-cholesterol (mmol/L), (**b**) portal vein serum triglycerides (mmol/L), (**c**) tail vein serum total cholesterol at week 3 (mmol/L), (**d**) tail vein serum LDL-cholesterol-to-HDL-cholesterol ratio at week 1, (**e**) aortic serum triglycerides-to-HDL-cholesterol ratio. Values are means ± SD. Mean values were significantly different from the control group: * $p < 0.05$ (one-way ANOVA and post-hoc Dunnett's test). MB, monobutyrin; LDL-cholesterol, low density lipoprotein-cholesterol; HDL-cholesterol, high-density lipoprotein-cholesterol; TG, triglycerides.

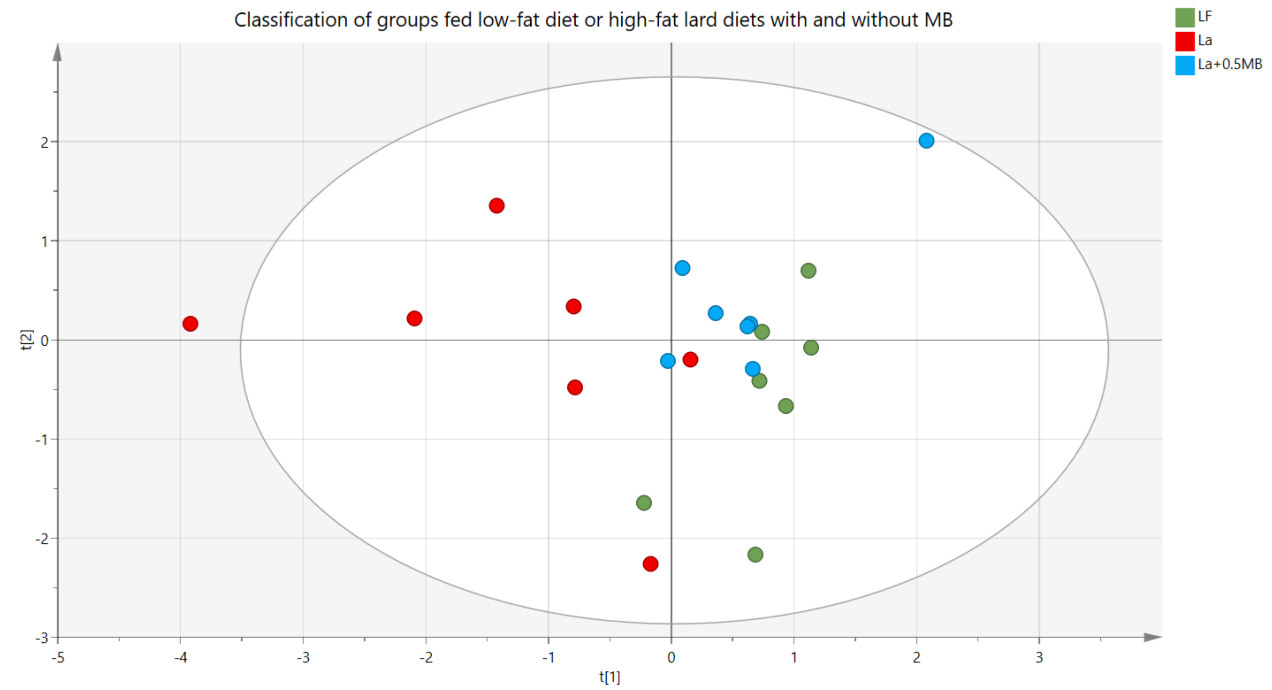

(a)

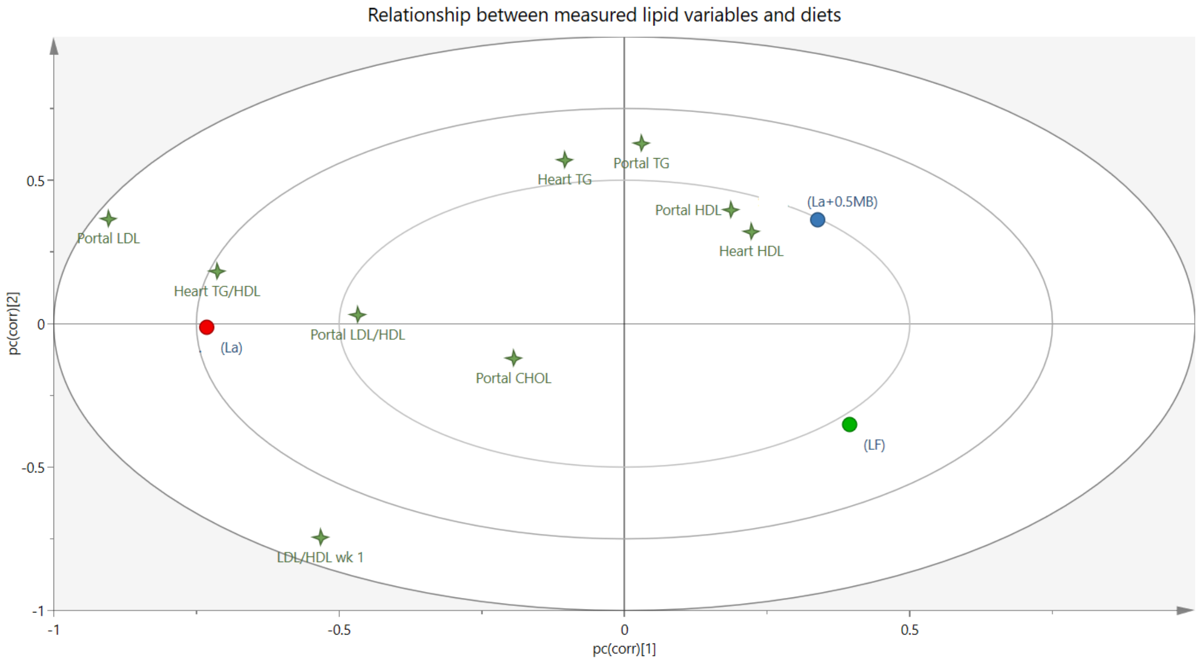

(b)

Figure 3. Effects of MB on lipids profiles in rats fed a low-fat (LF) diet, a high-fat control diet with lard (La), or the same diet supplemented with 0.5 MB g/100g (dry weight basis) (La + 0.5 MB) for 4 weeks. (**a**) Separation of the experimental groups, (b) relationship between measured lipid variables (4-point stars) and the experimental groups (circles). MB, monobutyrin; CHOL, cholesterol; TG, triglycerides; LDL, low-density lipoprotein-cholesterol; HDL, high-density lipoprotein-cholesterol.
